# Comparison of self-report and administrative data sources to capture health care resource use in people with chronic obstructive pulmonary disease following pulmonary rehabilitation

**DOI:** 10.1186/s12913-020-05920-0

**Published:** 2020-11-23

**Authors:** Chantal L. Grimwood, Anne E. Holland, Christine F. McDonald, Ajay Mahal, Catherine J. Hill, Annemarie L. Lee, Narelle S. Cox, Rosemary Moore, Caroline Nicolson, Paul O’Halloran, Aroub Lahham, Rebecca Gillies, Angela T. Burge

**Affiliations:** 1 Physiotherapy, The Alfred, PO Box 315, Prahran, VIC 3181 Australia; 2 La Trobe University Clinical School, Level 4 The Alfred Centre, 99 Commercial Road, Melbourne, VIC 3004 Australia; 3 Department of Allergy, Immunology and Respiratory Medicine, Monash University, Level 6, The Alfred Centre, 99 Commercial Rd, Melbourne, VIC 3004 Australia; 4 Institute for Breathing and Sleep, Level 5, Harold Stokes Building, Austin Health, PO Box 5555, Heidelberg, VIC 3084 Australia; 5 Department of Respiratory and Sleep Medicine, Austin Health, PO Box 5555, Heidelberg, VIC 3084 Australia; 6 Department of Medicine, The University of Melbourne, Parkville, VIC 3010 Australia; 7 The Nossal Institute for Global Health, The University of Melbourne, Parkville, VIC 3010 Australia; 8 Physiotherapy, Austin Health, PO Box 5555, Heidelberg, VIC 3084 Australia; 9 Physiotherapy, Monash University, Building B, McMahons Rd, Frankston, VIC 3199 Australia; 10 Public Health, La Trobe University, Bundoora, VIC 3086 Australia

**Keywords:** COPD, Health care utilisation, Self-report, Medical records, Hospitalisation, Accuracy, Diary

## Abstract

**Background:**

The optimal method to collect accurate healthcare utilisation data in people with chronic obstructive pulmonary disease (COPD) is not well established. The aim of this study was to determine feasibility and compare self-report and administrative data sources to capture health care resource use in people with COPD for 12 months following pulmonary rehabilitation.

**Methods:**

This is a secondary analysis of a randomised controlled equivalence trial comparing centre-based and home-based pulmonary rehabilitation. Healthcare utilisation data were collected for 12 months following pulmonary rehabilitation from self-report (monthly telephone questionnaires and diaries) and administrative sources (Medicare Benefits Schedule, medical records). Feasibility was assessed by the proportion of self-reports completed and accuracy was established using month-by-month and per participant comparison of self-reports with administrative data.

**Results:**

Data were available for 145/163 eligible study participants (89%, mean age 69 (SD 9) years, mean forced expiratory volume in 1 s 51 (SD 19) % predicted; *n* = 83 male). For 1725 months where data collection was possible, 1160 (67%) telephone questionnaires and 331 (19%) diaries were completed. Accuracy of recall varied according to type of health care encounter and self-report method, being higher for telephone questionnaire report of emergency department presentation (Kappa 0.656, *p* < 0.001; specificity 99%, sensitivity 59%) and hospital admission (Kappa 0.669, *p* < 0.001; specificity 97%, sensitivity 68%) and lower for general practitioner (Kappa 0.400, *p* < 0.001; specificity 62%, sensitivity 78%) and medical specialist appointments (Kappa 0.458, *p* < 0.001; specificity 88%, sensitivity 58%). A wide variety of non-medical encounters were reported (allied health and nursing) which were not captured in administrative data.

**Conclusion:**

For self-reported methods of healthcare utilisation in people with COPD following pulmonary rehabilitation, monthly telephone questionnaires were more frequently completed and more accurate than diaries. Compared to administrative records, self-reports of emergency department presentations and inpatient admissions were more accurate than for general practitioner and medical specialist appointments.

**Trial registration:**

NCT01423227 at clinicaltrials.gov

**Supplementary Information:**

The online version contains supplementary material available at 10.1186/s12913-020-05920-0.

## Background

Healthcare utilisation refers to individual or population use of healthcare services. Depending on the context and information of interest, data regarding healthcare utilisation can be collected by administrative or self-reported methods, with relative advantages and limitations to both approaches. In addition to informing healthcare planning and policy, data accuracy is a key consideration in clinical trials where healthcare utilisation may be a study outcome or used to underpin economic evaluations of healthcare [1].

Administrative sources of healthcare utilisation identify routinely-collected data from hospitals, governments or insurance companies. Administrative sources collect comprehensive data on easily identifiable health care contacts (e.g. hospital admissions) and are generally considered as the standard for reporting utilisation [2]. The steady growth in adoption of electronic health records [3] and activity-based funding [4] in developed countries have been associated with improved operational performance and efficient documentation [5]; however, high-quality administrative data are unavailable for most of the world’s population, particularly in low- and middle-income countries where system funding, infrastructure and capacity can preclude cohesive documentation [3]. Depending on the specific source, access to administrative data can also be limited by regulations, costly to procure and restricted to the scope of services offered/funded by the organisation [6]. Accuracy of administrative data may be compromised in the context of manifold sources incorporating multiple healthcare payers and providers [7–9]. This can pose a particular challenge when individuals access numerous services for chronic and/or multiple conditions over repeat visits [10] as is the case for people with chronic obstructive pulmonary disease (COPD) who access a wide range and large volume of healthcare services, with healthcare utilisation up to 3.4 fold higher than their healthy peers [11].

An alternative approach to collecting healthcare utilisation data is self-report, which may be administered in person, via telephone or diaries [7] and is commonly used in large population-based studies [12, 13]. In contrast to administrative sources, self-report data may incorporate a broader range of healthcare services, such as non-subsidised allied health visits, and additional details, such as reason for the appointment or associated out of pocket expenses, which are not comprehensively documented elsewhere [14]. However, collection of self-report data can also be time-consuming and expensive to undertake. Depending on the ability of the participant to recollect past events, data accuracy may also be subject to bias from poor recall [2] and telescoping [8]. Recall bias occurs when there is a discrepancy in the recollection of the information that may involve forgetting an encounter (memory decay) or recalling an encounter that did not occur [15, 16]. Forward or backward telescoping incorrectly places an event within or outside of the recall period [7]. Greater inaccuracy has been associated with longer recall periods [8, 15–17] as well as participant features such as age [18, 19] and frequency of healthcare encounters [20] which are also commonly demonstrated in people with COPD.

Substantial variations between self-report healthcare utilisation and administrative records have been demonstrated in a range of populations [21–24]. Generally, agreement between sources is better for events such as hospitalisations or emergency department (ED) presentations relative to outpatient and general practitioner (GP) appointments, tests and types of imaging [2, 7, 9]. However, the optimal method to collect accurate healthcare utilisation data in people with COPD is not well established. The aim of this study was to determine feasibility and accuracy of self-report methods compared to administrative records for healthcare utilisation in people with COPD for 12 months following pulmonary rehabilitation in a country with well-established systems for gathering administrative data.

## Methods

This study presents a secondary analysis of a randomised controlled equivalence trial conducted at two tertiary public hospitals in Melbourne, Australia in which participants were recruited from pulmonary rehabilitation waiting lists between 21 October 2011 and 3 April 2014**.** The trial received institutional ethics approval and was prospectively registered (clinicaltrials.gov, NCT01423227). Participants provided written informed consent, as well as separate consent to obtain data for federal government funded health care (Medicare Benefits Schedule, MBS). No compensation was provided for study participation.

### Australian health care system context

Health care in Australia is delivered by a mix of public and private sector entities, with various components funded by government, private health insurers and out-of-pocket payments by individuals [25]. Funded by taxpayers, Medicare is the universal public health insurance scheme that provides the majority of Australian health care services [26]. The MBS is a key component, incorporating subsidised services that includes medical consultation fees and diagnostic tests. Individual-level data can be requested for health research, subject to privacy and confidentiality criteria, administrative fees and processing time following study completion [27]. Australian hospitals receive activity based funding, whereby funding is based on the number and type of patients treated and therefore requires rigorously collated data collection [28]. In Australia the majority of non-medical health care encounters occur outside hospitals and are not subsidised by the MBS, so these were not able to be comprehensively identified from administrative records.

### Clinical trial

The trial protocol and clinical outcomes have been published in accordance with CONSORT guidelines [29, 30]. In summary, 166 individuals with stable COPD were randomised to centre or home-based pulmonary rehabilitation including exercise training and self-management education. Participants in the home-based program received an initial physiotherapy home visit to establish goals and supervise their first exercise session. Following this, participants received seven once-weekly telephone calls from a physiotherapist, using structured modules and motivational interviewing. The centre-based program consisted of twice-weekly supervised outpatient group sessions. Study measurements were recorded at baseline, at the end of pulmonary rehabilitation and 12 months following program completion.

### Data collection

Collected participant characteristics at baseline were: age (years); sex (male/female); intervention group (home-based/centre-based); symptoms as assessed by the chronic respiratory disease questionnaire (CRQ; score); disease severity as assessed by the forced expiratory volume in 1 s (FEV_1_; % predicted); functional exercise tolerance as assessed by the distance walked in 6-min walk test (6MWD; metres); and comorbidity test [COTE] index) [31]. Healthcare utilisation data were collected for each participant for 12 months following completion of pulmonary rehabilitation.

Administrative data were sourced from the MBS and hospital records. The MBS data included outpatient services including GP visits, medical specialist appointments and eligible allied health encounters. Data were also collected from medical and finance department records from the healthcare service where pulmonary rehabilitation was undertaken. Electronic medical records were screened for encounters within each participant’s 12-month study period, including clinical notes and correspondence detailing service access. Data included outpatient appointments (medical specialists, other health care professionals), ED presentations and hospital admissions.

Self-report data were collected on a monthly basis by telephone and written diary (preceding 4-week period). Participants were contacted by telephone by the same research team member each month where possible (call time approximately 5–10 min). Using a questionnaire, participants were prompted to report healthcare visits (GP, medical specialist, other healthcare professionals) in any environment, as well as ED presentations and hospital admissions. Where participants reported a hospital admission within a different health service and confirming documentation was unavailable, that hospital was contacted to confirm admission details. Participants were also sent monthly diaries for the 12-month period and were encouraged to use the diary to record unsupervised exercise sessions as well as health care encounters during the monthly telephone calls; diaries were returned at their final study assessment.

### Statistical analysis

Analysis was undertaken using IBM SPSS Statistics (v25). Descriptive statistics are presented according to type of data and distribution. For this study, administrative data were regarded as the most accurate measure of healthcare utilisation.

Five types of health care encounters were analysed: GP visits; medical specialist appointments; ED presentations; hospital admissions; and visits to other health care professionals. Records for each month of self-report data (telephone questionnaire, diary) were compared to relevant administrative records (GP visits; medical specialist appointments; ED presentations; hospital admissions) to determine accuracy according to whether any health care encounter occurred (Y/N), any encounter was reported (Y/N) and total number of encounters reported. Records for each participant were classified according to the number of monthly records that were available and correct, total numbers of encounters correctly reported and number of encounters missed. For medical specialist, ED presentations and hospital admissions, telescoping was also identified (reported events outside the recall time frame). Comprehensive administrative sources for visits to other health care professionals were not available so only descriptive data were presented.

Feasibility of each self-reported data source was determined by the proportion of completed telephone questionnaires and returned diaries. The relationship between the number of completed telephone questionnaires and returned diaries with participant characteristics at baseline was examined using linear regression. The covariates were: age; sex; intervention group; dyspnoea domain of the CRQ (CRQ-D); FEV_1_; 6MWD; and COTE index.

For each type of health care encounter, agreement between administrative and self-reported data was demonstrated using the Kappa coefficient (dichotomised: event reported/not reported) and correlation using the Spearman coefficient (r_s_) (number of events). Matching of administrative data and self-reported data (data sources match) was calculated (numerator was the number of months self-report data matched administrative data, denominator was the total number of completed monthly telephone questionnaires or diaries). Concordance between self-reported and administrative data was reflected by calculation of specificity (numerator was the number of months with no reported health care encounters in both self-report and administrative data, denominator the number of months with no health care encounters according to administrative records) and sensitivity (numerator was the number of months where health care encounters matched in self-report and administrative data, denominator was the number of months with health care encounters according to administrative records).

Linear regression was used to examine the relationship between participant characteristics at baseline (covariates as per previous analyses) and the accuracy of self-report data (telephone questionnaires and returned diaries). Accuracy was quantified as: (i) Months correct – months in which at least one health care encounter had occurred and was correctly identified; (ii) Months with missed visits – months in which a health care encounter had occurred but was missing in self-reported data; (iii) Months with wrong/extra visits – months in which a health care encounter was reported that had not occurred, or was additional to that documented in administrative data; and (iv) Months with matched visits – self-report data matched administrative records on all criteria, including presence/absence of visits and the correct number of visits. For encounter**s** with other health care professionals, descriptive data were presented.

## Results

In total, 163 participants from the clinical trial were eligible for this analysis; three participants died before completion of pulmonary rehabilitation. Of these, 145 participants (89%) consented to MBS data collection and were therefore included in this study; characteristics were similar between participants who did and did not consent to MBS data collection (Table 1). No participants withdrew from this study, and no participants were withdrawn on the basis of inability to contact. The occasions on which participants could not be contacted were counted as months where self-report data could have been obtained but telephone questionnaires were not completed.

**Table 1:** Participant characteristics at baseline

	Participants included in analysis *n* = 145 (89%)	Participants not included in analysis *n* = 18 (11%)	p
Centre-based intervention group, n (%)	73 (50%)	11 (61%)	0.389
Age, years	69 (9)	72 (12)	
Male, n (%)	83 (57%)	14 (78%)	0.094
FEV_1_, % predicted	49 [36–63]	55 [41–60]	
COPD-specific comorbidity test (COTE) score ≥ 4, n (%)	28 (19%)	4 (22%)	0.769
6-min walk distance, metres	423 [325–508]	410 [353–452]	
Chronic Respiratory Questionnaire, score			
Dyspnoea	15 [11–18]	14 [9–18]	
Fatigue	15 [11–18]	14 [11–16]	
Emotional Function	32 [26–39]	27 [23–37]	
Mastery	18 [15–24]	16 [13–20]	

Data are mean (SD) or median [IQR] unless indicated. Significance *p* < 0.05. Included participants were those who consented to collection of administrative data (Medicare Benefits Schedule)

*COPD* chronic obstructive pulmonary disease, *FEV*_*1*_ forced expiratory volume in 1 s, *MBS* Medicare Benefits Schedule

There was a total of 1725 months where self-report data could be obtained (excluded 15 months where three participants were deceased); 1160 (67%) telephone questionnaires were completed and 331 (19%) diaries were returned. Eight participants completed no telephone questionnaires and 94 participants did not return any diaries. Five admissions were reported outside of catchment areas without confirmatory documentation in the medical record and were subsequently confirmed with relevant facilities.

Administrative data demonstrated that the greatest number of encounters were GP visits, followed by medical specialist visits, ED presentations and hospital admissions. Only 2% of participants had no GP visits, whilst > 50% of participants had an ED presentation and/or hospital admission. Overall, a higher proportion of administrative health care encounters were reported by participants in telephone questionnaires relative to diary data but reporting varied according to type of health care encounter (Table 2). Incremental increases in baseline dyspnoea score were associated with a small but statistically significant reduction in the number of telephone questionnaires completed and increase in the number of returned diaries. Incremental increases in baseline functional exercise tolerance were also associated with a small but statistically significant increase in the number of returned diaries (Supplementary Table 1).

**Table 2:** Proportion of health care encounters reported according to type of health care

	GP visit	Medical specialist appointment	ED presentation	Hospital admission
Participants with no encounters, n (%)	3 (2%)	23 (16%)	81 (56%)	77 (53%)
Total encounters (administrative), n	1926	872	166	153
Telephone questionnaires				
Total reportable encounters^a^, n	1283	607	120	122
Correctly reported, n (%)	765 (60%)	298 (49%)	59 (49%)	72 (59%)
Diaries				
Total reportable encounters^a^, n	266	151	15	23
Correctly reported, n (%)	164 (62%)	52 (34%)	2 (13%)	7 (30%)

^a^Reportable encounters = where a participant had the opportunity to report the encounter. For example, if a phone call was not completed in a given month, then any health care utilisation in that month was unable to be reported

*ED* emergency department, *GP* general practitioner

Agreement between sources was highest for hospital admissions (Kappa 0.6, *p* < 0.001) and lowest for ED presentations (Kappa 0.2, *p* < 0.001). Matching of administrative data and self-reported telephone questionnaire data was highest for ED presentations and hospital admissions (both 95%; Kappa 0.7, *p* < 0.001), and lowest for GP visits (72%; Kappa 0.4, *p* < 0.001). Specificity was higher for ED presentations and hospital admissions (telephone questionnaires 99, 97%; diaries 99, 99%; respectively). Sensitivity was lower for medical specialist appointments (telephone questionnaires 58%; diaries 43%) and ED presentations (telephone questionnaires 59%; diaries 13%; Table 3).

**Table 3:** Agreement between administrative and self-reported data according to type of health care

	Telephone questionnaires				Diaries			
	GP visit	Medical specialist appointment	ED presentation	Hospital admission	GP visit	Medical specialist appointment	ED presentation	Hospital admission
Agreement, Kappa	0.4 *p* < 0.001	0.5 *p* < 0.001	0.7 *p* < 0.001	0.7 *p* < 0.001	0.5 *p* < 0.001	0.3 *p* < 0.001	0.2 *p* < 0.001	0.6 *p* < 0.001
Correlation, Spearman	0.4 *p* < 0.001	0.5 *p* < 0.001	N/A	N/A	0.6 *p* < 0.001	0.4 *p* < 0.001	N/A	N/A
Data sources match^a^	834/1160 (72%)	902/1160 (78%)	1104/1160 (95%)	1097/1160 (95%)	255/331 (77%)	241/331 (73%)	315/331 (95%)	316/331 (96%)
Specificity^b^	269/437 (62%)	669/758 (88%)	1044/1058 (99%)	1024/1052 (97%)	124/149 (83%)	192/216 (89%)	313/315 (99%)	305/308 (99%)
Sensitivity^c^	565/723 (78%)	233/402 (58%)	60/102 (59%)	73/108 (68%)	131/182 (72%)	49/115 (43%)	2/16 (13%)	11/23 (48%)

Data are n (%) unless indicated, significance *p* < 0.05. 1160 telephone questionnaires from 137 participants; 331 diaries from 51 participants

*ED* emergency department, *GP* general practitioner, *N/A* not calculated due to low number

^a^numerator = the number of months self-report data matched administrative data, denominator = the total number of completed monthly telephone questionnaires or diaries

^b^numerator = the number of months with no reported health care encounters in both self-report and administrative data, denominator = the number of months with no health care encounters according to administrative records

^c^numerator = the number of months where health care encounters matched in self-report and administrative data, denominator = the number of months with health care encounters according to administrative records

With regard to accuracy of telephone questionnaires, there was a statistically significant relationship between increases in COTE index score (i.e. more comorbidities) and an increase in the number of months in which at least one GP visit was correctly identified. However, an increase in COTE index score was also associated with an increase in the number of months where visits were missing from telephone-reported data, (GP visits, ED presentations and hospital admissions), and where wrong/extra ED presentations and hospital admissions were reported (Supplemental Tables 2, 4 and 5). Increases in 6MWD were associated with an increase in the number of months when telephone-reported data matched administrative records for both GP visits and specialist encounters, and a decrease in the number of months with missed specialist encounters (Supplemental Tables 2 and 3).

With regard to accuracy of returned diaries, higher CRQ-D scores were associated with a decrease in the number of months in which the number of GP visits and specialist encounters were correctly reported (Supplemental Tables 2 and 3).

Encounters with 11 other types of health care professionals were identified from participant self-report, with few of these identifiable in administrative data (Table 4). Higher numbers of non-medical encounters were reported in monthly telephone questionnaires compared to hospital records.

**Table 4:** Number of health care encounters with other health care professionals identified in administrative and self-reported data for individual participants over the 12-months following pulmonary rehabilitation

	Administrative data		Self-report data	
	Medicare benefits schedule	Medical record	Phone calls	Diaries
Podiatrist	0–7	0–14	0–17	0–2
Nurse	0–4	0–48	0–77	0–5
Optometrist	0–3	Nil	0–1	0–2
Dentist	0–5	0–2	0–7	0–1
Dietician	0–3	0–10	0–4	Nil
Physiotherapist	0–5	0–36	0–70	0–20
Psychologist	0–10	0–9	0–4	0–5
Occupational therapist	0–2	0–4	0–2	Nil
Speech pathologist	Nil	0–3	Nil	Nil
Social worker	Nil	0–1	Nil	Nil
Pharmacist	Nil	0–5	Nil	Nil
Total number	261	665	858	72

Data are the range of number of encounters per participant

## Discussion

The results of this study demonstrated that monthly telephone questionnaires were more frequently completed than diaries and therefore present a more feasible means of self-report healthcare utilisation data collection in people with COPD over 12 months following pulmonary rehabilitation. Relative to administrative data, self-report data for hospitalisation and ED presentation have high specificity but modest sensitivity, with less accurate recall of GP and medical specialist visits. Analyses of participant characteristics indicated that diaries may be more feasible in patients who are more symptomatic. Non-medical encounters (nursing and allied health) were reported commonly by patients in monthly telephone questionnaires. The feasibility findings have important implications in the context of emerging COVID 19-related precautions. In many parts of the world where face-to-face household surveys have been the mainstay for data collection to date [12, 13], these results help inform resource re-allocation decisions as data collection processes adapt.

Self-report data underestimated healthcare utilisation relative to administrative data. Frequent encounters, such GP and medical specialist appointments, were less accurately reported than less frequent and possibly more salient events, such as ED attendances and hospital admissions. This is in accordance with previous studies in older people [19], people with chronic health conditions [22] and following inpatient rehabilitation [16]. The high specificity and lower sensitivity demonstrated for ED attendances and hospital admissions indicates that overestimation is unlikely using self-report methods for these events. The more accurate reports of ‘non-use’ rather than ‘use’ of these services indicates the important role of administrative data sources to avoid underestimation of utilisation.

The observed under-reporting of GP visits over 12 months was consistent with earlier findings [7] and with earlier work demonstrating a relationship between increased encounter frequency and under-reporting [8]. In this study, the most frequent encounters were GP appointments, ranging up to 62 visits with a mean 13 visits over the 12-month period, which was much higher than in other studies of participants with COPD (mean 7.9 [32]) and other chronic conditions (mean 3.8 [22]). There is no research identifying the optimal recall period for self-report healthcare utilisation data in people with COPD. Participants in this study were contacted each month, with the intention of avoiding the bias associated with longer recall periods [33, 34]. These results, in the context of a relatively short recall period, would indicate telescoping of visits (forward or backwards) rather than memory decay as the source of inaccuracy. Additionally, less accurate self-report of healthcare utilisation by participants with a higher COTE index was demonstrated, which is an important consideration in people with COPD who frequently demonstrate comorbidities that have considerable clinical and economic consequences [35].

Conflicting evidence for the impact of self-report relative to administrative sources on estimates of cost-effectiveness has been demonstrated [33, 34]. Whilst GP visits may be the most common type of encounter, hospital admissions and ED presentations form the most costly component of care and demonstrated higher levels of agreement between sources. More work is required to further elucidate the optimal use of these sources.

A wide variety of healthcare practitioners accessed by individuals with COPD was highlighted in this study. Aside from the four key encounter types that were investigated, interactions with 11 other healthcare professionals were self-reported over the 12-month period following pulmonary rehabilitation. In our study, the most commonly utilised ‘other’ healthcare professionals were nurses (up to 77 visits) and physiotherapists (up to 70 visits). A previous study investigated recall of respiratory nurse encounters and physiotherapy visits by people with COPD and demonstrated a high degree of agreement for physiotherapists (r_s_ > 0.8) and substantial agreement for respiratory nurses (r_s_ > 0.6) [34]. However, study design may have been a key factor, with protocol-driven regular appointments with physiotherapists and respiratory nurses allowing a fixed administrative source for data comparison. In this study, telephone questionnaire data did provide additional data on non-medical encounters beyond the scope of accessed administrative sources. However, we were unable to draw any conclusions regarding the accuracy of these data as care provided by the majority of these healthcare practitioners are not MBS-subsidised and are frequently accessed outside the hospital system, so are not comprehensively documented in any single administrative source.

### Limitations

These results relate to people with COPD who have undertaken pulmonary rehabilitation, and may not be relevant to other populations. Due to study design and availability of resources, the sample size was small and limited to two tertiary health services in Melbourne. Study participation required access to a telephone for intervention delivery, and therefore results may not be generalisable to more disadvantaged groups. Another important limitation is the lack of a true ‘gold standard’ against which to compare the accuracy of participant self-reported healthcare contacts. However, the administrative data were drawn from a health system in a developed country with well-developed electronic record keeping and reimbursement systems related to hospital and GP contacts, so for these outcomes the administrative data are likely to provide the most comprehensive coverage. The telephone questionnaire and diary were specifically designed for this group and were not alidated measurement tools. The 19% diary return rate may have been attributable to our method of collection (collecting all diaries at 12-month follow-up), participant burden to regularly record data for 12 months, or participant perception of data utility (in addition to telephone questionnaires). Regardless, it was much lower compared to another study in COPD that reported that 83% of monthly healthcare utilisation booklets were returned over a 2-year study period [34] which may also reflect other participant, study or cultural variations.

## Conclusion

In people with COPD following pulmonary rehabilitation, monthly telephone questionnaires were more frequently and accurately completed than diaries as self-reported methods of collecting healthcare utilisation data. Compared to administrative records, ED presentations and hospital admissions were more accurately self-reported than GP and medical specialist appointments, with high specificity but more modest sensitivity. Self-report methods identified a broad range of healthcare contacts outside the scope of administrative records. This study highlighted important considerations in the use of self-report and administrative methods of healthcare utilisation data collection in people with COPD over 12 months, particularly for application to broader healthcare planning and healthcare expenditure purposes.

## Supplementary Information

**Additional file 1: Supplementary Table 1.** Participant features associated with feasibility of self-reported healthcare utilisation data. **Supplementary Table 2.** Participant features associated with accurate reporting of general practitioner visits. **Supplementary Table 3.** Participant features associated with accurate reporting of medical specialist appointments. **Supplementary Table 4.** Participant features associated with accurate reporting of emergency department presentations. **Supplementary Table 5.** Participant features associated with accurate reporting of hospital admissions.

## Data Availability

The datasets used and/or analysed during the current study are available from the corresponding author on reasonable request.

## Abbreviations

COPD: Chronic obstructive pulmonary disease
ED: Emergency department
FEV_1_: Forced expiratory volume in 1 s
HADS: Hospital anxiety and depression scale
MBS: Medicare Benefits Schedule
6MWT: Six-minute walk test
SF-36: 36-Item Short Form Health Survey version 2
