# Supplementary material for: Comparison of self-report and administrative data sources to capture health care resource use in people with chronic obstructive pulmonary disease following pulmonary rehabilitation
Source: BMC Health Serv Res. 2020 Nov 23;20:1061. doi: 10.1186/s12913-020-05920-0 (PMC7682690; doi:10.1186/s12913-020-05920-0)
Supplement: Supplementary file 1 — Additional file 1: Supplementary Table 1. Participant features associated with feasibility of self-reported healthcare utilisation data. Supplementary Table 2. Participant features associated with accurate reporting of general practitioner visits. Supplementary Table 3. Participant features associated with accurate reporting of medical specialist appointments. Supplementary Table 4. Participant features associated with accurate reporting of emergency department presentations. Supplementary Table 5. Participant features associated with accurate reporting of hospital admissions. [file 12913_2020_5920_MOESM1_ESM.docx]

**Supplementary Table 1** Participant features associated with feasibility of self-reported healthcare utilisation data

|  | Number of telephone questionnaires | | Number of returned diaries | |
| --- | --- | --- | --- | --- |
| (Constant) | 9.8 | (3.8, 15.8) | -7.4 | (-14.2, -0.7) |
| Age (years) | 0.0 | (-0.1, 0.1) | 0.1 | (-0.0, 0.1) |
| Female (vs. male) | -0.4 | (-1.6, 0.7) | 0.9 | (-0.4, 2.2) |
| Home-based intervention group (vs. centre-based) | -0.4 | (-1.5, 0.7) | 0.2 | (-1.1, 1.4) |
| CRQ-D (score) | -0.13 | (-0.23, -0.02)* | 0.12 | (0.01, 0.24)* |
| FEV_1_ (% predicted) | 0.01 | (-0.03, 0.04) | -0.00 | (-0.04, 0.03) |
| 6MWD (metres) | 0.001 | (-0.005, 0.006) | 0.006 | (0.000, 0.012)* |
| COTE index (score) | -0.1 | (-0.4, 0.1) | -0.1 | (-0.3, 0.2) |

β (95%CI) = unstandardized coefficient B (95% confidence interval). *statistical significance

CRQ-D = dyspnoea domain of the chronic respiratory disease questionnaire; FEV_1_ = forced expiratory volume in one second; 6MWD = distance walked on six-minute walk test; COTE = comorbidity test.

**Supplementary Table 2** Participant features associated with accurate reporting of general practitioner visits

|  | Telephone questionnaires | | | | Returned diaries | | | |
| --- | --- | --- | --- | --- | --- | --- | --- | --- |
|  | Months correct (≥1 visit) | Months with missed visit(s) | Months with wrong/extra visit(s) | Months with matched visit(s) | Months correct (≥1 visit) | Months with missed visit(s) | Months with wrong/extra visit(s) | Months with matched visit(s) |
| (Constant) | 1  (-7, 8) | 7  (-1, 15) | 0  (-8, 8) | -1  (-4, 3) | 7  (-2, 16) | -2  (-11, 7) | 1  (-3, 6) | 0  (-6, 6) |
| Number of telephone questionnaires/returned diaries | 0.6  (0.3, 0.8) | 0.25  (-0.02, 0.52) | 0.2  (-0.1, 0.4) | 0.3  (0.2, 0.4) | 0.5  (0.3, 0.7) | 0.2  (-0.1, 0.4) | 0.2  (0.1, 0.3) | 0.4  (0.2, 0.5) |
| Age (years) | 0.0  (-0.1, 0.1) | 0.0  (-0.1, 0.1) | 0.06  (-0.02, 0.15) | -0.03  (-0.06, 0.01) | 0.0  (-0.1, 0.1) | 0.08  (-0.03, 0.18) | 0.0  (-0.1, 0.1) | -0.03  (-0.09, 0.04) |
| Female (vs. male) | -0.1  (-1.4, 1.3) | -0.7  (-2.2, 0.8) | -0.9  (-2.3, 0.6) | 0.1  (-0.5, 0.8) | -0.8  (-2.3, 0.8) | 0.0  (-1.6, 1.6) | -1.1  (-1.9, -0.3)* | 0.2  (-0.8, 1.3) |
| Home-based intervention group (vs. centre-based) | -0.4  (-1.7, 0.9) | -1.9  (-3.4, -0.5)* | -1.0  (-2.4, 0.4) | 0.4  (-0.2, 1.1) | -0.8  (-2.3, 0.6) | -0.8  (-2.3, 0.7) | -0.3  (-1.1, 0.5) | 0.3  (-0.7, 1.2) |
| CRQ-D (score) | 0.0  (-0.1, 0.2) | 0.0  (-0.2, 0.1) | 0.0  (-0.2, 0.1) | 0.0  (-0.1, 0.1) | -0.142  (-0.279, -0.004)* | 0.0  (-0.1, 0.2) | 0.0  (-0.1, 0.1) | 0.0  (-0.1, 0.1) |
| FEV_1_ (% predicted) | 0.03  (-0.01, 0.06) | 0.01  (-0.03, 0.05) | -0.01  (-0.05, 0.03) | -0.0179  (-0.0356, -0.0001)* | 0.00  (-0.04, 0.04) | -0.02  (-0.06, 0.02) | -0.01  (-0.03, 0.01) | 0.01  (-0.02, 0.03) |
| 6MWD (metres) | -0.0070  (-0.0136, -0.0004)* | -0.0071  (-0.0145, 0.0003) | 0.002  (-0.005, 0.009) | 0.004  (0.001, 0.007)* | -0.001  (-0.009, 0.006) | -0.001  (-0.009, 0.006) | 0.002  (-0.002, 0.005) | 0.002  (-0.003, 0.006) |
| COTE index (score) | 0.3  (0.1, 0.6)* | 0.286  (0.004, 0.568)* | -0.1  (-0.4, 0.2) | -0.2  (-0.3, -0.1)* | 0.1  (-0.2, 0.4) | 0.1  (-0.2, 0.5) | 0.1  (-0.1, 0.2) | -0.23  (-0.43, -0.02)* |

Data are β (95%CI) = unstandardized coefficient B (95% confidence interval). *statistical significance

CRQ-D = dyspnoea domain of the chronic respiratory disease questionnaire; FEV_1_ = forced expiratory volume in one second; 6MWD = distance walked on six-minute walk test; COTE = comorbidity test.

**Supplementary Table 3** Participant features associated with accurate reporting of medical specialist appointments

|  | Telephone questionnaires | | | | Returned diaries | | | |
| --- | --- | --- | --- | --- | --- | --- | --- | --- |
|  | Months correct (≥1 appointment) | Months with missed appointment(s) | Months with wrong/extra appointment(s) | Months with matched appointment(s) | Months correct (≥1 appointment) | Months with missed appointment(s) | Months with wrong/extra appointment(s) | Months with matched appointment(s) |
| (Constant) | 3  (-2, 9) | 7  (0, 13) | 0  (-3, 4) | -2  (-7, 3) | 1  (-5, 7) | 2  (-7, 10) | -1  (-7, 4) | 0  (-7, 6) |
| Number of telephone questionnaires/returned diaries | 0.3  (0.1, 0.4) | 0.23  (0.02, 0.44) | 0.11  (-0.01, 0.23) | 0.6  (0.4, 0.7) | 0.3  (0.1, 0.4) | 0.21  (-0.01, 0.43) | 0.11  (-0.03, 0.25) | 0.6  (0.4, 0.8) |
| Age (years) | -0.05  (-0.10, 0.01) | -0.04  (-0.11, 0.03) | 0.01  (-0.03, 0.05) | 0.00  (-0.05, 0.06) | 0.0  (-0.1, 0.1) | 0.0  (-0.1, 0.1) | 0.0  (-0.1, 0.1) | 0.0  (-0.1, 0.1) |
| Female (vs. male) | 0.3  (-0.7, 1.3) | -0.4  (-1.6, 0.8) | 0.1  (-0.6, 0.8) | -0.1  (-1.0, 0.8) | 0.1  (-0.9, 1.1) | -0.6  (-2.1, 0.8) | 0.1  (-0.8, 1.1) | 0.2  (-1.0, 1.4) |
| Home-based intervention group (vs. centre-based) | 0.1  (-0.9, 1.0) | -0.6  (-1.8, 0.6) | -0.1  (-0.7, 0.6) | 0.0  (-0.9, 0.9) | 0.0  (-0.9, 1.0) | 0.6  (-0.8, 2.0) | 0.0  (-0.9, 0.9) | -0.2  (-1.3, 0.9) |
| CRQ-D (score) | -0.07  (-0.16, 0.02) | 0.0  (-0.1, 0.1) | 0.0  (-0.1, 0.1) | 0.0  (-0.1, 0.1) | -0.10  (-0.19, -0.02)* | 0.0  (-0.1, 0.1) | 0.0  (-0.1, 0.1) | 0.07  (-0.04, 0.18) |
| FEV_1_ (% predicted) | 0.026  (-0.002, 0.054) | 0.02  (-0.02, 0.05) | 0.00  (-0.02, 0.02) | -0.01  (-0.04, 0.01) | 0.01  (-0.01, 0.04) | 0.02  (-0.02, 0.06) | -0.01  (-0.03, 0.02) | -0.02  (-0.05, 0.01) |
| 6MWD (metres) | -0.003  (-0.007, 0.002) | -0.008  (-0.013, -0.002)* | -0.002  (-0.005, 0.002) | 0.006  (0.002, 0.010)* | 0.000  (-0.004, 0.005) | -0.0073  (-0.0141, -0.0005)* | 0.002  (-0.003, 0.006) | 0.004  (-0.002, 0.009) |
| COTE index (score) | 0.1  (-0.1, 0.3) | 0.1  (-0.1, 0.3) | 0.0  (-0.1, 0.1) | -0.14  (-0.31, 0.02) | 0.0  (-0.2, 0.2) | 0.1  (-0.2, 0.4) | 0.1  (-0.1, 0.3) | -0.1  (-0.3, 0.1) |

Data are β (95%CI) = unstandardized coefficient B (95% confidence interval). *statistical significance

CRQ-D = dyspnoea domain of the chronic respiratory disease questionnaire; FEV_1_ = forced expiratory volume in one second; 6MWD = distance walked on six-minute walk test; COTE = comorbidity test.

**Supplementary Table 4** Participant features associated with accurate reporting of emergency department presentations

|  | Telephone questionnaires | | | | Returned diaries | | | |
| --- | --- | --- | --- | --- | --- | --- | --- | --- |
|  | Months correct (≥1 presentation) | Months with missed presentation(s) | Months with wrong/extra presentation(s) | Months with matched presentation(s) | Months correct (≥1 presentation) | Months with missed presentation(s) | Months with wrong/extra presentation(s) | Months with matched presentation(s) |
| (Constant) | 1  (-1, 3) | 1  (-1, 2) | 2  (0, 3) | -3  (-6, 0) | 2  (-5, 9) | -10  (-47, 28) | 4  (-9, 17) | -1  (-4, 2) |
| Number of telephone questionnaires/returned diaries | 0.053  (-0.004, 0.109) | 0.04  (-0.02, 0.09) | 0.03  (-0.01, 0.07) | 0.9  (0.8, 1.0) | 0.08  (-0.03, 0.18) | -0.2  (-0.7, 0.4) | 0.1  (-0.1, 0.3) | 0.9  (0.8, 1.0) |
| Age (years) | 0.00  (-0.02, 0.02) | 0.00  (-0.02, 0.02) | -0.019  (-0.033, -0.005)* | 0.01  (-0.02, 0.05) | 0.0  (-0.1, 0.1) | 0.1  (-0.4, 0.5) | 0.0  (-0.2, 0.1) | 0.01  (-0.02, 0.04) |
| Female (vs. male) | -0.1  (-0.5, 0.2) | 0.1  (-0.3, 0.4) | -0.1  (-0.3, 0.2) | 0.0  (-0.6, 0.6) | -0.2  (-1.3, 0.9) | 2.3  (-3.5, 8.0) | -0.7  (-2.7, 1.3) | 0.0  (-0.5, 0.5) |
| Home-based intervention group (vs. centre-based) | -0.2  (-0.5, 0.1) | 0.0  (-0.3, 0.3) | 0.0  (-0.2, 0.3) | 0.4  (-0.2, 0.9) | -0.5  (-1.2, 0.1) | -0.7  (-4.2, 2.8) | 0.1  (-1.1, 1.3) | -0.2  (-0.6, 0.3) |
| CRQ-D (score) | -0.02  (-0.05, 0.01) | -0.01  (-0.04, 0.02) | 0.01  (-0.01, 0.04) | 0.02  (-0.03, 0.07) | 0.0  (-0.1, 0.1) | 0.2  (-0.4, 0.8) | -0.1  (-0.3, 0.1) | 0.03  (-0.01, 0.08) |
| FEV_1_ (% predicted) | 0.00  (-0.01, 0.01) | 0.00  (-0.01, 0.01) | -0.003  (-0.010, 0.003) | 0.00  (-0.02, 0.02) | -0.02  (-0.06, 0.01) | 0.04  (-0.14, 0.21) | -0.01  (-0.07, 0.05) | 0.00  (-0.01, 0.01) |
| 6MWD (metres) | -0.001  (-0.002, 0.001) | -0.0013  (-0.0029, 0.0002) | -0.001  (-0.002, 0.001) | 0.0025  (-0.0003, 0.0053) | 0.001  (-0.003, 0.004) | 0.003  (-0.015, 0.020) | -0.002  (-0.008, 0.004) | 0.001  (-0.001, 0.004) |
| COTE index (score) | 0.01  (-0.04, 0.07) | 0.10  (0.04, 0.16)* | 0.06  (0.02, 0.11)* | -0.12  (-0.22, -0.01)* | 0.0  (-0.1, 0.1) | -0.1  (-0.6, 0.4) | 0.15  (-0.02, 0.32) | -0.06  (-0.15, 0.04) |

Data are β (95%CI) = unstandardized coefficient B (95% confidence interval). *statistical significance

CRQ-D = dyspnoea domain of the chronic respiratory disease questionnaire; FEV_1_ = forced expiratory volume in one second; 6MWD = distance walked on six-minute walk test; COTE = comorbidity test.

**Supplementary Table 5** Participant features associated with accurate reporting of hospital admissions

|  | Telephone questionnaires | | | | Returned diaries | | | |
| --- | --- | --- | --- | --- | --- | --- | --- | --- |
|  | Months correct (≥1 admission) | Months with missed admission(s) | Months with wrong/extra admission(s) | Months with matched admission(s) | Months correct (≥1 admission) | Months with missed admission(s) | Months with wrong/extra admission(s) | Months with matched admission(s) |
| (Constant) | 2  (-1, 4) | 1  (-2, 4) | 0  (-3, 2) | -4  (-6, -1) | 3  (-2, 9) | 0  (-5, 5) | -1  (-4, 2) | -1  (-4, 3) |
| Number of telephone questionnaires/returned diaries | 0.111  (0.005, 0.218) | 0.0  (-0.1, 0.1) | 0.10  (0.01, 0.19) | 0.9  (0.8, 1.0) | 0.17  (0.02, 0.32) | 0.0  (-0.1, 0.2) | 0.06  (-0.02, 0.13) | 1.0  (0.9, 1.0) |
| Age (years) | 0.00  (-0.04, 0.03) | -0.01  (-0.04, 0.03) | -0.01  (-0.04, 0.02) | 0.02  (-0.01, 0.05) | 0.0  (-0.1, 0.1) | 0.0  (-0.1, 0.1) | 0.02  (-0.03, 0.06) | 0.00  (-0.04, 0.04) |
| Female (vs. male) | -0.1  (-0.7, 0.5) | 0.2  (-0.4, 0.8) | -0.1  (-0.5, 0.4) | 0.1  (-0.4, 0.6) | -0.5  (-1.9, 0.8) | 0.4  (-0.8, 1.5) | 0.1  (-0.6, 0.7) | -0.1  (-0.7, 0.5) |
| Home-based intervention group (vs. centre-based) | -0.1  (-0.6, 0.5) | -0.1  (-0.7, 0.4) | 0.2  (-0.3, 0.6) | 0.2  (-0.4, 0.7) | -0.2  (-1.2, 0.9) | -0.4  (-1.2, 0.5) | -0.1  (-0.6, 0.4) | 0.1  (-0.5, 0.6) |
| CRQ-D (score) | -0.02  (-0.09, 0.04) | 0.0  (-0.1, 0.1) | 0.04  (-0.01, 0.09) | 0.02  (-0.03, 0.07) | 0.0  (-0.1, 0.1) | 0.0  (-0.1, 0.1) | 0.0  (-0.1, 0.1) | 0.01  (-0.04, 0.07) |
| FEV_1_ (% predicted) | 0.00  (-0.02, 0.02) | 0.01  (-0.01, 0.03) | -0.01  (-0.02, 0.01) | 0.01  (-0.01, 0.02) | -0.02  (-0.06, 0.01) | 0.0299  (-0.0001, 0.0599)* | -0.01  (-0.02, 0.01) | 0.00  (-0.01, 0.02) |
| 6MWD (metres) | -0.002  (-0.005, 0.001) | -0.002  (-0.005, 0.001) | 0.001  (-0.002, 0.003) | 0.0023  (-0.0002, 0.0049) | 0.002  (-0.002, 0.007) | -0.0041  (-0.0079, -0.0002)* | 0.000  (-0.002, 0.003) | 0.000  (-0.003, 0.003) |
| COTE index (score) | 0.0  (-0.1, 0.1) | 0.14  (0.04, 0.24)* | 0.13  (0.05, 0.21)* | -0.2  (-0.3, -0.1)* | -0.1  (-0.2, 0.1) | 0.13  (-0.03, 0.29) | 0.0  (-0.1, 0.1) | 0.0  (-0.2, 0.1) |

Data are β (95%CI) = unstandardized coefficient B (95% confidence interval). *statistical significance

CRQ-D = dyspnoea domain of the chronic respiratory disease questionnaire; FEV_1_ = forced expiratory volume in one second; 6MWD = distance walked on six-minute walk test; COTE = comorbidity test.
